# Amikacin and bacteriophage treatment modulates outer membrane proteins composition in *Proteus mirabilis* biofilm

**DOI:** 10.1038/s41598-020-80907-9

**Published:** 2021-01-15

**Authors:** Agnieszka Maszewska, Magdalena Moryl, Junli Wu, Bin Liu, Lu Feng, Antoni Rozalski

**Affiliations:** 1 Department of Biology of Bacteria, Institute of Microbiology, Biotechnology and Immunology, Faculty of Biology and Environmental Protection, University of Lodz, Banacha 12/16, 90-237 Lodz, Poland; 2 TEDA Institute of Biological Sciences and Biotechnology, Nankai University, Tjanjin, People’s Republic of China

**Keywords:** Antimicrobial resistance, Biofilms, Bacteriophages

## Abstract

Modification of outer membrane proteins (OMPs) is the first line of Gram-negative bacteria defence against antimicrobials. Here we point to *Proteus mirabilis* OMPs and their role in antibiotic and phage resistance. Protein profiles of amikacin (AMKrsv), phage (Brsv) and amikacin/phage (AMK/Brsv) resistant variants of *P. mirabilis* were compared to that obtained for a wild strain. In resistant variants there were identified 14, 1, 5 overexpressed and 13, 5, 1 downregulated proteins for AMKrsv, Brsv and AMK/Brsv, respectively. Application of phages with amikacin led to reducing the number of up- and downregulated proteins compared to single antibiotic treatment. Proteins isolated in AMKrsv are involved in protein biosynthesis, transcription and signal transduction, which correspond to well-known mechanisms of bacteria resistance to aminoglycosides. In isolated OMPs several cytoplasmic proteins, important in antibiotic resistance, were identified, probably as a result of environmental stress, e.g. elongation factor Tu, asparaginyl-tRNA and aspartyl-tRNA synthetases. In Brsv there were identified: NusA and dynamin superfamily protein which could play a role in bacteriophage resistance. In the resistant variants proteins associated with resistance mechanisms occurring in biofilm, e.g. polyphosphate kinase, flagella basal body rod protein were detected. These results indicate proteins important in the development of *P. mirabilis* antibiofilm therapies.

## Introduction

*Proteus mirabilis,* Gram-negative rods, are involved in 65–95% of catheter associated urinary tract infections, which are related with the biofilm formation. Because of *P. mirabilis* ability to synthesize urease, biofilm is often encrusted with struvite and apatite crystals, which provides an excellent niche for bacterial cells, protecting them from external environmental factors, e.g. antibiotics^1,2^. Concentrations of drugs for biofilm eradication are 1000–1500 times higher than doses needed to eliminate their planktonic counterparts^3^.

Bacterial resistance to antimicrobials is related with the biofilm lifestyle. The extracellular matrix (ECM) could bind molecules (e.g. phages, antibiotics) and prevent or delay their diffusion to the receptors on target cells. Slow metabolic activity of bacterial cells and the presence of dormant spore-like cells, called persisters in the biofilm structure are also involved in the mechanisms of resistance to antibiotics^4^. Furthermore, Gram-negative bacteria have evolved many more resistance factors, e.g. the outer membrane (OM), which provide an extra layer of protection. OM is a combination of a highly hydrophobic lipid bilayer and various proteins. Modifications in the components influence the selectivity of the barrier and antimicrobials sensitivity. Accordingly, new methods are being developed to eliminate biofilm e.g. combined use of an antibiotic and a bacteriophage^5^. Bacteriophages (phages), which infect and kill bacteria, have several features that make them good candidates for anti-biofilm agents. Phages can penetrate the biofilm by water channels reaching the cells located in its deeper layers and can express enzymes that degrade the ECM. By damaging the biofilm, phages facilitate access of antibiotics to bacteria living in the biofilm. Persister cells can also be infected by bacteriophages^6,7^. The potential of a combined use of bacteriophages and antibiotics in biofilms eradication has been previously tested and a significant biofilm disruption and reduction of the resistant variants in *Klebsiella pneumoniae* or *E. coli* formation has been observed^5,8–10^.

Because of their polycationic nature, aminoglycosides, bind to the anionic compounds of the bacterial cell wall. In Gram-negative bacteria it could be a lipopolysaccharide, phospholipids and outer membrane proteins (OMPs). This process is energy-independent and leads to an increase in permeability and to the so called “self-promoted uptake”, which allows the antibiotic molecules to gain access into the periplasmic space. Next, in an energy-dependent process, aminoglycosides reach the cytoplasm, where they contribute to the formation of mistranslated proteins. It results in a disruption of the cytoplasmic membrane integrity and allows penetration of a large amount of the antibiotic into the bacterial cell, which leads to the formation of a large amount of abnormal proteins and, consequently, cell death^11^. The first line of bacterial defence against AGs may result in the modification of cell membrane permeability (limiting the uptake of antibiotics) or in activating diverse efflux pumps (removing the antibiotic from the cell)^12^. The importance of changing the composition of bacterial OMPs in the acquisition of resistance to antibiotics has been demonstrated for *Pseudomonas aeruginosa*, *Escherichia coli*, *Helicobacter pylori* and *Vibrio cholerae*^13,14^. Unfortunately, the modification of OMPs in Gram-negative bacteria could result in the loss or alteration of the phage receptor, which prevents phage adsorption and leads to the inhibition of infection^6^.

In the present study, amikacin or/and phage *P. mirabilis* resistant variants were isolated from biofilm and, next, the OMPs profiles of the resistant variants were compared to that obtained from a wild strain (a variant sensitive to the tested factors). The differences in OMPs expression could help to indicate proteins which play a crucial role in *P. mirabilis* antibiotic and/or phage resistance.

## Results

### Determination of the amikacin concentration for the selection of *P. mirabilis* resistant variants

An appropriate concentration of amikacin for the selection of resistant variants was determined using MIC (Minimum Inhibitory Concentration), MBC (Minimum Bactericidal Concentration) and MBIC (Minimum Biofilm Inhibitory Concentration) methods. The MIC and MBC value for the wild strains of *P. mirabilis* was 16 µg/mL, with the exception of strain 3059, where MBC reached 32 µg/mL. The MBIC values were determined colorimetrically by an MTT viability assay and defined as the amikacin concentration causing a 50% decrease in the metabolic activity of cells in the biofilm^15^. Amikacin MBICs for the biofilms of strains C41, C77, 1281 and 3059 were 8, 32, 32 and 64 µg/mL, respectively. Based on the obtained results, the amikacin concentration of 16 µg/mL was used for the selection of resistant variants.

### *P. mirabilis* biofilm sensitivity to amikacin and/or bacteriophage

**Figure 1:**
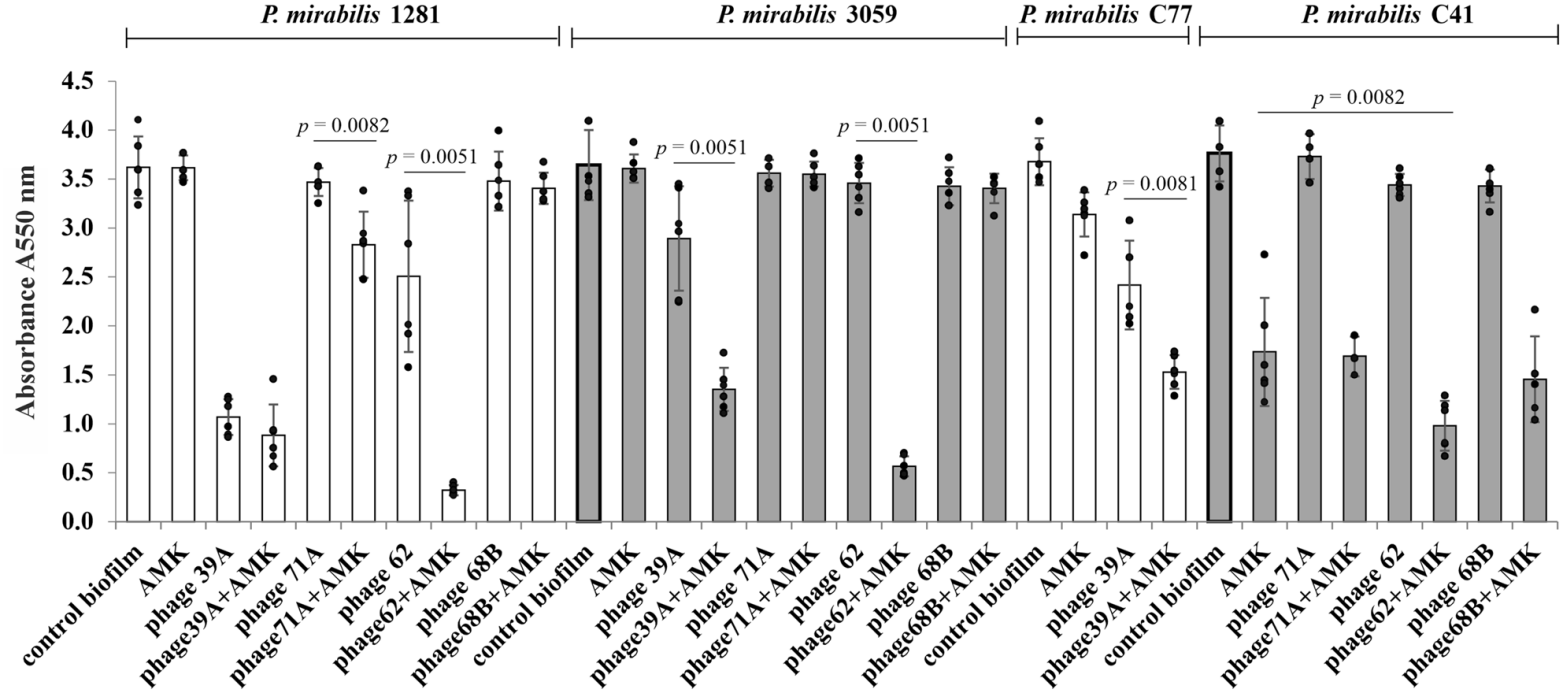
The intensity of *P. mirabilis* biofilms after 24-h amikacin and/or bacteriophage treatment. Control biofilm—biofilm incubated in medium without phage or/and amikacin; AMK—biofilm treated with amikacin (16 μg/mL); phage 39A, phage 71A, phage 62, or phage 68B—biofilm treated with appropriate phage (10^7^ PFU/mL); phage39A + AMK, phage71A + AMK, phage62 + AMK, or phage68B + AMK—biofilm treated with combination of amikacin (16 μg/mL) and appropriate phage (10^7^ PFU/mL). Bars—arithmetic mean calculated for the results (n = 6); error bars, mean ± standard deviation; black circles—individual results. Mann–Whitney U-test were used for statistical data analysis. *P* values < 0.05 were considered significant.

The effect of phages 39A, 71A, 62 or 68B and amikacin, used separately and in combination, on 24-h biofilms of *P. mirabilis* strains was evaluated by determination of percentage reduction in the absorbance values in relation to the biofilm untreated with antimicrobials (Fig. 1). Amikacin did not reduce the biofilm of strains 1281 and 3059, however, it caused a 15 ± 6% and 54 ± 15% decrease in the biofilm viability in strains C77 and C41, respectively. Phages 68B and 71A did not affect the tested biofilms, whereas phage 39A destroyed the biofilms of strains 1281, C77 and 3059 in the range of 21 ± 15% to 70 ± 5%. Only the biofilm of strain 1281 was susceptible to phage 62, and its metabolic activity was reduced by 31 ± 21%. While testing the influence of the amikacin-phage mixture on the *P. mirabilis* biofilms, a synergistic effect of the used antimicrobials on the *P. mirabilis* biofilms was observed in 50% of the cases (Fig. 1). These differences were statistically significant P value was between 0.0051 and 0.0082 (Mann–Whitney U-test). The combined anti-biofilm effect of amikacin and phages was considered to be a synergistic when it was stronger compared to the best acting agent used alone.

The combination of amikacin-phage 62 was the most effective in the destruction of the *P. mirabilis* 1281 and 3059 strains in the tested biofilms. The metabolic activity of biofilms treated with this mixture was lower by about 91 ± 1% and 84 ± 3%, respectively than that obtained for control biofilms of those strains. The antibiotic and phage 62 used alone did not affect these biofilms significantly.

### Selection of *P. mirabilis* bacteriophages or/and amikacin resistant variants

AMK, phage and AMK/phage resistant variants were selected from *P. mirabilis* biofilms using firstly liquid (LSM) and afterwards solid selective media (SSM), which contained the anti-biofilm agents (Table 1). In most cases the variants isolated from AMK-treated biofilms presented weak or medium growth. On the contrary, during the isolation of resistant variants, where phages acted as a selection factor, in almost all cases an intensive growth of bacteria was observed on selective media. Interestingly, no or weak growth on selective media was noted during the isolation of amikacin and bacteriophage resistant variants.

**Table 1:** *Proteus mirabilis* amikacin resistant variant—AMKrsv (A), bacteriophage resistant variant—Brsv (B) and amikacin and bacteriophage resistant variant—AMK/Brsv (C) selection for outer membrane proteins analysis.

*P. mirabilis* strain	Selective factor	Growth intensity		RTD (PFU/mL)		MIC in phage broth (µg/mL)	
		LSM	SSM	WT	Resistant variants	WT	Resistant variants
**(A) amikacin resistant variant—AMKrsv**							
1281	Amikacin	1	1	NA	NA	16	128
3059		0/2	1	NA	NA	16	256
C77		1	3	NA	NA	16	64
C41		0/1	1	NA	NA	16	256
**(B) bacteriophage resistant variant—Brsv**							
1281	B39A	2	3	10^7^	R	NA	NA
	B71A	3	3	10^6^	10^8^	NA	NA
	B62	3	3	10^6^	10^8^	NA	NA
	B68B	2	3	10^5^	R	NA	NA
3059	B39A	3	3	10^7^	R	NA	NA
	B71A	3	3	10^7^	10^8^	NA	NA
	B62	3	3	10^6^	10^8^	NA	NA
	B68B	2	3	10^5^	10^5^	NA	NA
C77	B39A	3	0	10^7^	R	NA	NA
C41	B71A	3	1	10^8^	10^8^	NA	NA
	B62	3	3	10^6^	10^8^	NA	NA
	B68B	1	3	10^5^	R	NA	NA
**(C) amikacin and bacteriophage resistant variant—AMK/Brsv**							
1281	B39A/AMK	0	0	10^7^	R	16	128
	B71A/AMK	0	2	10^6^	10^8^		256
	B62/AMK	0	0	10^6^	R		128
	B68B/AMK	0	0	10^5^	R		128
3059	B39A/AMK	0	1	10^7^	R	16	128
	B71A/AMK	1	2	10^7^	R		256
	B62/AMK	1	3	10^6^	R		64
	B68B/AMK	0	1	10^5^	R		128
C77	B39A/AMK	0	1	10^7^	R	16	64
C41	B71A/AMK	0	1	10^8^	–	16	–
	B62/AMK	0	0	10^6^	–		–
	B68B/AMK	0	0	10^5^	–		–

R—phage resistant, Growth intensity on LSM and SSM: 0—no growth, 1—weak growth, 2—medium growth, 3—intensive growth, RTD (Routine Test Dilution)—a phage suspension at a concentration, which produces complete lysis of a bacterial lawn.

*AMK* amikacin, *B* bacteriophage, *WT* wild type *Proteus mirabilis* 3059 strain, *LSM* liquid selective medium containing phages 1 × 10^7^ PFU/mL or amikacin (16 µg/mL) or both factors, *SSM* solid selective medium: phage agar containing phages 1 × 10^7^ PFU/mL for Brsv selection and Mueller–Hinton plate containing amikacin (16 µg/mL) or antibiotic and phages for selection of AMKrsv or AMK/Brsv, respectively.

To confirm the selection of bacteriophage and/or amikacin resistant variants, the routine dilution test (RTD) was conducted, or MIC values of isolated variants and wild-type strain were determined. The RTD values for wild strains were 10^5^–10^8^ depending on the bacteriophage and the bacterial strain (Table 1 B, C). The RTD values determined for isolated phage or amikacin and bacteriophage resistant variants were equal to or 10–100-fold higher than the value designated for the wild type strain. For some resistant variants, RTD values could not be determined, suggesting that these isolates were phage resistant (R). The MICs of AMK and AMK/phage resistant variants were determined, and a 4–16-fold increase in the MIC value of resistant variants compared to the wild type strain was observed (Table 1 A, C).

Based on the obtained results, amikacin (AMKrsv), phage (Brsv) and amikacin and phage (AMK/Brsv) resistant variants of *P. mirabilis* 3059 were selected for comparative analysis of outer membrane proteins. The biofilm formed by this strain alone was resistant to AMK and phage 62 used separately, and was destroyed at 84 ± 3% by these agents used in combination. What is more, phage and AMK/phage resistant variants exhibited intensive growth on SSM.

### Proteomic analysis of OMPs of *P. mirabilis* wild strain, amikacin and/or bacteriophage resistant variants

**Figure 2:**
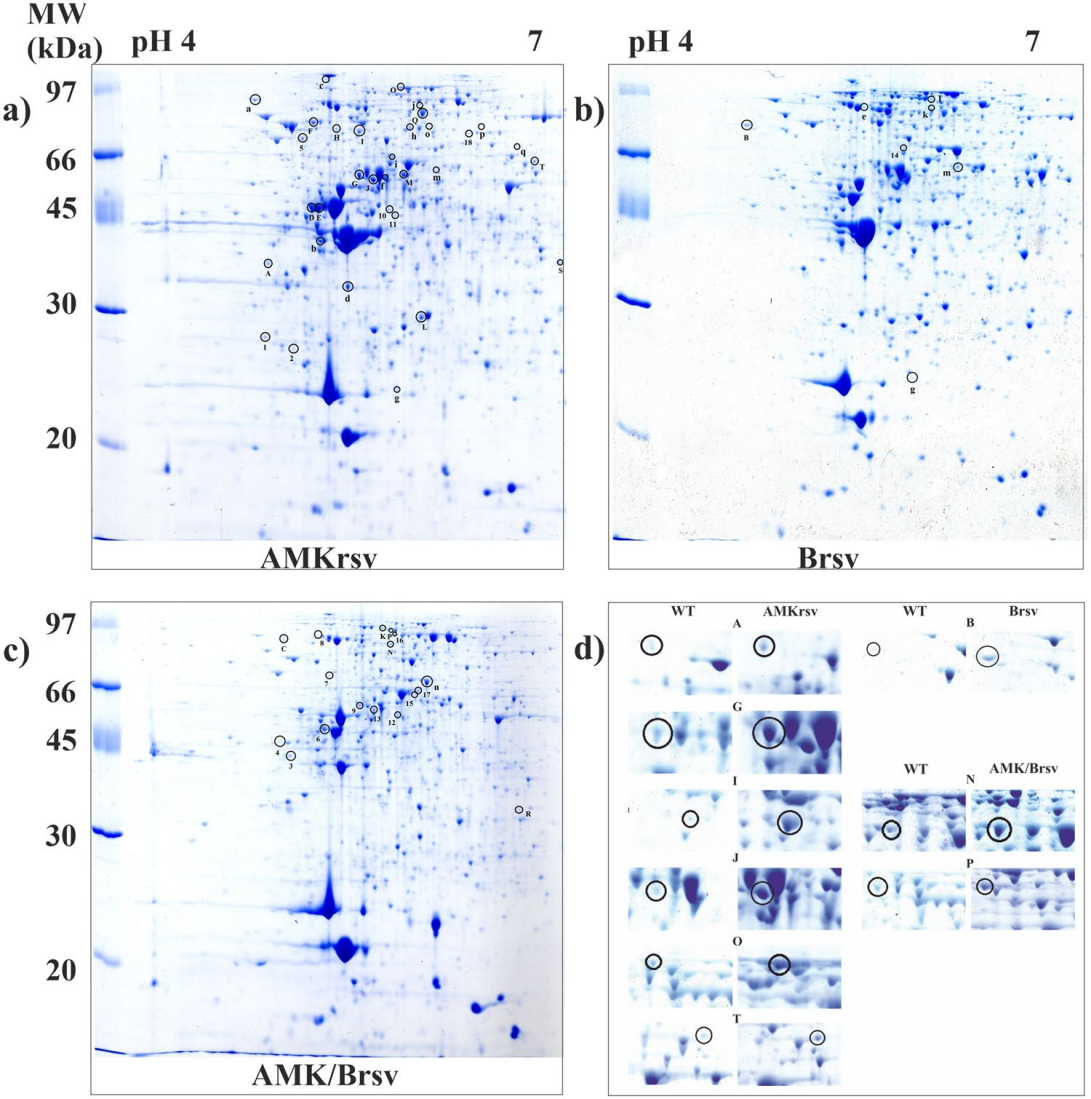
2D profiles of OMPs *Proteus mirabilis* 3059: (**a**) amikacin resistant variant (AMKrsv), (**b**) bacteriophage resistant variant (Brsv), (**c**) amikacin and bacteriophage resistant variant (AMK/Brsv) and (**d**) magnified regions of selected overexpressed protein spots in the resistant variants. Proteins were stained with Coomassie Brilliant Blue G-250, gels were scanned using a Power-Look 1000 (UMAX Technologies Inc., Dallas, TX), and were analysed by PDQuest version 7.3.0 (Bio-Rad Laboratories). Spots marked with numbers indicate proteins which were detected only in studied resistant variants. Spots marked with capital letters or lowercase letters correspond to proteins with higher or lower level of expression in the resistant variants, respectively. Description of the proteins are included in Tables 2, 3,4. WT—wild type *Proteus mirabilis* 3059. Spots identification: A—elongation factor Tu [*Proteus vulgaris*]; B—transcription termination factor NusA; G—asparaginyl-tRNA synthetase; I—aspartyl-tRNA synthetase; J—ATP-dependent protease ATP-binding subunit HslU; N—ClpA protein; O—polyphosphate kinase; P—flagella basal body rod protein; T—glycerol-3-phosphate dehydrogenase.

Triton-X 100 insoluble proteins were isolated from the biofilm of a wild strain and all resistant variants of *P. mirabilis* 3059. 2DE profiles were conducted in triplicates for all variants, next the composite images (master gels) were prepared to compare the protein profiles (Fig. 2, full-length gels are presented in Supplementary Fig. 1). The obtained gels were analysed by the PD Quest Advanced software, version 7.3.0 (Bio-Rad Laboratories). Over 500 protein spots were resolved for each of isolated variants. 2DE analysis revealed differentially expressed proteins in wild *P. mirabilis* 3059 strain and resistant variants, which could be related in resistance to tested antimicrobial agents. All spots were excised from the gels, most of which (76%) were successfully identified by MALDI-TOF MS/MS.

During protein analysis the identified proteins were subdivided into three sections: proteins occurring only in individual resistant variants and absent in the wild type strain (1); proteins with significantly increased (2) or decreased (3) intensity in the resistant variants. Cut limit ≥ 3-fold changes in spots intensity between the wild type strain and resistant variants were considered as significant. Tables 2, 3 and 4 list selected and identified proteins and summarise their most important features and functions. The spots on the gels corresponding to these proteins are encircled in Fig. 2. Magnified regions of selected overexpressed protein spots in the resistant variants are shown in Fig. 2d.

**Table 2:** Description of proteins specific to studied *P. mirabilis* 3059 amikacin resistant variant (AMKrsv) identified by MALDI TOF MS/MS.

Amikacin resistant variants							
Spot symbol	Protein identification	Protein MW (Da)	Protein PI	Peptide count	Protein score	Protein score C.I. %	Function
**Proteins detected only in the resistant variant**							
1	AGAP012287-PA	59,651	8.72	10	81.4	96	Me
2	N-Acetylgalactosamine-specific phosphotransferase enzyme iib component1	18,634	7.82	6	73.7	75	Me
5	30S ribosomal protein S1	61,293	4.90	11	102.0	100	Me
11	Glutamyl-tRNA synthetase	53,548	5.40	8	92.2	100	Me
18	Glycyl-tRNA synthetase subunit beta	78,693	5.94	10	68.3	13	Me
10	Membrane fusion protein AcrA	42,287	7.70	12	136.0	100	Tr
**Proteins with a higher level of expression**							
Q	DNA-binding transcriptional regulator GlcC	28,859	8.55	11	75.0	81	In
H	Protein kinase C inhibitor 1 (pkcI)	13,623	6.97	7	88.9	99	Me
O	Polyphosphate kinase	84,830	6.71	7	76.7	87	Me
A	Elongation factor Tu [*Proteus vulgaris*]	30,104	4.69	14	191.0	100	Me
D	Elongation factor Tu [*Proteus vulgaris*]	30,104	4.75	16	184.0	100	Me
E	Elongation factor Tu [*Proteus vulgaris*]	30,104	4.69	14	155.0	100	Me
F	Elongation factor G	77,820	5.04	16	111.0	100	Me
G	Asparaginyl-tRNA synthetase	52,297	5.05	14	117.0	100	Me
I	Aspartyl-tRNA synthetase	66,529	5.18	17	118.0	100	Me
S	Ribosomal protein L14	12,990	10.67	6	70.6	49	Me
J	ATP-dependent protease ATP-binding subunit HslU	49,855	5.09	11	77.0	88	Me
T	Glycerol-3-phosphate dehydrogenase	56,828	6.59	11	96.1	100	Me
M	Adenylosuccinate synthetase	47,439	5.38	12	110.0	100	Me
L	Chain A, structure of M182t mutant of Tem-1 beta-lactamase	28,973	5.46	18	243.0	100	Vi
**Proteins with a lower level of expression**							
c	rpoB gene product	150,862	5.23	13	96.5	100	In
f	DNA helicase	109,241	6.57	15	76.5	87	In
g	DNA gyrase subunit A	92,435	5.43	11	72.9	70	In
a	P-512 [*Borrelia turicatae*]	269,373	4.77	23	72.7	68	Me
b	rplW gene product	12,330	9.93	6	74.8	81	Me
d	MoxR-like ATPase	35,714	4.85	9	74.1	77	Me
h	sdhA gene product (succinate dehydrogenase flavoprotein subunit)	64,803	5.65	9	95.7	100	Me
i	Aldose 1-epimerase	37,986	6.39	8	80.1	94	Me
m	sucA gene product (2-oxoglutarate dehydrogenase E1 component)	106,057	5.74	11	77.7	90	Me
p	3-Dehydroquinate synthase [*Thermococcus kodakarensis* KOD1]	37,286	5.31	9	77.3	89	Me
j	ABC transporter ATP-binding protein	34,105	5.81	6	72.5	67	Tr
o	Fimbrial outer membrane usher protein	97,049	6.86	17	131.0	100	Vi
q	Chain A, formation of A tyrosyl radical intermediate in proteus mirabilis catalase by directed muta	55,558	6.12	16	213.0	100	B

Protein function: *In* information pathways, *Me* metabolism, *Tr* transport, *Vi* virulence, *Ch* chaperone. Spots marked with numbers indicate proteins which were detected only in the studied resistant variants. Spots marked with capital or lowercase letters correspond to proteins with a higher or lower level of expression in the resistant variants, respectively.

**Table 3:** Description of proteins specific to studied *P. mirabilis* 3059 bacteriophage resistant variant (Brsv) identified by MALDI TOF MS/MS.

Bacteriophage resistant variant							
Spot symbol	Protein identification	Protein MW (Da)	Protein PI	Peptide count	Protein score	Protein score C.I. %	Function
**Proteins detected only in the resistant variant**							
14	Negative elongation factor B	68,293	6.30	7	70.9	52	In
**Proteins with a higher level of expression**							
B	Transcription termination factor NusA	54,884	4.51	13	98.9	100	In
**Proteins with a lower level of expression**							
g	DNA gyrase subunit A	92,435	5.43	11	72.9	70	In
e	MoxR-like ATPase	35,714	4.85	8	79.4	93	Me
k	Dynamin family protein	97,035	5.11	14	90.3	99	Me
m	sucA gene product (2-oxoglutarate dehydrogenase E1 component)	106,057	5.74	11	77.7	90	Me
l	Protein disaggregation chaperone	95,793	5.57	13	96.9	100	Ch

Protein function: *In* information pathways, *Me* metabolism, *Tr* transport, *Vi* virulence, *Ch* chaperone. Spots marked with numbers indicate proteins which were detected only in the studied resistant variants. Spots marked with capital or lowercase letters correspond to proteins with a higher or lower level of expression in the resistant variants, respectively.

**Table 4:** Description of proteins specific to studied *P. mirabilis* 3059 amikacin and bacteriophage resistant variant (AMK/Brsv) identified by MALDI TOF MS/MS.

Amikacin and bacteriophage resistant variants							
Spot symbol	Protein identification	Protein MW (Da)	Protein PI	Peptide count	Protein score	Protein score C.I. %	Function
**Proteins detected only in the resistant variant**							
16	Transcriptional regulator of sucrose operon	36,977	6.41	9	83.0	97	In
7	Malate dehydrogenase	63,416	5.60	12	111.0	100	Me
3	Elongation factor Tu [*Morganella morganii* subsp. sibonii]	30,156	4.80	12	136.0	100	Me
4	Elongation factor Tu [*Proteus vulgaris*]	30,104	4.70	12	149.0	100	Me
6	Elongation factor Tu [*Proteus vulgaris*]	30,104	4.70	15	161.0	100	Me
8	Elongation factor G	77,820	5.00	12	94.3	100	Me
15	Glycerol kinase	56,069	5.80	12	120.0	100	Me
17	Glycerol kinase	56,069	5.84	10	85.3	98	Me
13	gnd gene product (6-phosphogluconate dehydrogenase)	51,563	5.10	13	103.0	100	Me
9	ATP-dependent protease ATP-binding subunit HslU	49,895	5.20	14	101.0	100	Ch
12	t-Complex protein 1, beta subunit, putative	57,431	5.80	10	71.6	59	Ch
**Proteins with a higher level of expression**							
C	Chromosomal replication initiation protein	52,191	6.63	9	71.8	61	In
K	Gyrase B	46,206	8.49	12	109.0	100	In
P	Flagella basal body rod protein	26,566	5.16	7	68.7	21	Vi
R	Hydrolyase, tartrate beta subunit/fumarate domain protein, Fe-S type	20,269	8.73	5	79.9	94	Vi
N	ClpA protein	95,441	5.4	17	138.0	100	Ch
**Proteins with a lower level of expression**							
n	Phosphoenolpyruvate carboxykinase	59,610	5.3	9	77.0	88	Me

Protein function: *In* information pathways, *Me* metabolism, *Tr* transport, *Vi* virulence, *Ch* chaperone. Spots marked with numbers indicate proteins which were detected only in the studied resistant variants. Spots marked with capital or lowercase letters correspond to proteins with a higher or lower level of expression in the resistant variants, respectively.

Most of the proteins (63%) which were isolated from the resistant variants play a role in bacterial metabolism, e.g. protein, purine, carbohydrate, polyphosphate, glycerol, lipid biosynthesis, in tricarboxylic acid cycle or in carbohydrate degradation. Proteins involved in the information pathways, virulence or acting as chaperones were also identified.

In the amikacin resistant variant (AMKrsv) there were found 33 proteins with changes in their expression level, seven proteins in the bacteriophage resistant variant (Brsv), whereas for the amikacin and bacteriophage resistant variant (AMK/Brsv) 17 such proteins were identified.

There were found 6 proteins expressed only in the AMK resistant variant (Table 2, Fig. 2a). That group included e.g. membrane fusion protein AcrA and glutamyl-tRNA synthetase, which could play an important role in amikacin resistance. The largest group (14 members) are proteins that were overexpressed in the AMK-resistant variant. The most important overexpressed proteins could be divided into two groups. The first one consisted of the proteins involved in the mechanisms of Gram-negative bacteria resistance to aminoglycosides. This group included, e.g. the elongation factor Tu, asparaginyl-tRNA and aspartyl-tRNA synthetase, adenylosuccinate synthetase, ribosomal protein L-14, ATP-dependent protease and ATP-binding subunit Hsl. The second group consisted of the proteins associated with the resistance mechanisms occurring in sessile forms during biofilm formation and contained: polyphosphate kinase (PPK), glycerol-3-phosphate dehydrogenase (GlpD). In AMKrsv there were also isolated 13 proteins with a lower level of expresion, e.g. MoxR-like ATPase, SucA gene product.

In Brsv one protein—negative elongation factor B was expressed only in that variant, and one protein—the transcription termination factor NusA was overexpressed (Table 3, Fig. 2b). Five proteins were downregulated. The most important ones are: MoxR-like ATPase, SucA gene product, dynamin superfamily protein. Bacteria in the deeper layers of the biofilm structure are characterized by slow growth and reduced metabolism, therefore in this study the same proteins were identified in different resistant variants: AMKrsv and Brsv.

In the amikacin and bacteriophage resistant variant (AMK/Brsv) there were detected 11 proteins with the expression only in that variant, for example: elongation factor Tu or ATP-dependent protease ATP-binding subunit HslU, which could be involved in the resistance to amikacin (Table 4, Fig. 2c). There were found 5 overexpressed proteins: chromosomal replication initiation protein, gyrase B, hydrolyase, tartrate beta subunit/fumarate domain protein, Fe-S type and ClpA protein and flagella basal body protein (FlgB) associated in bacterial strategy against antimicrobials. One protein-phosphoenolopyruvate carboxykinase (PEPCK) was downregulated in AMK/Brsv, which is closely related to aminoglicoside resistance.

Summarizing, in all resistant variants there were identified 20 overexpressed proteins, 14 for AMKrsv, 1 for Brsv and 5 for AMK/Brsv. There were 19 proteins with a lower expression in all resistant isolates. Similarly, to the overexpressed proteins, most downregulated proteins were identified for AMKrsv (13). Only 5 and 1 proteins were found in Brsv and AMK/Brsv, respectively.

## Discussion

Resistance of bacterial biofilms to commonly used drugs^3^ forced the search for other anti-biofilm agents and the testing of alternative therapies. A combined use of bacteriophage and an antibiotic is suggested as a more effective method of biofilm eradication, where fewer resistant variants are isolated compared to single therapies^16,17^. Comeau et al.^18^ in their study on phage and beta-lactam and quinolone antibiotics stated that phage-antibiotic synergy stimulate virulent phage growth. An increased burst size along with a reduced latent period of phages was observed. Dual treatment was significantly more efficient in biofilm eradication compared to the single therapies. Furthermore, decreasing the therapeutic dose of an antibiotic used with phages minimizes adverse side effects of the antibiotics in vivo^17^. The outer membrane proteins (OMPs) in Gram-negative bacteria pose a barrier for antimicrobials and appropriate modifications of lipids and proteins in their structure are an important bacterial resistance mechanism. The role of OMPs downregulation in the development of *Pseudomonas aeruginosa*, *E. coli* and *Vibrio cholera* antibiotic resistance was described earlier^13^. Bacteriophages use some OMPs as receptors during adsorption to the host cell. The first of phage resistant mechanisms involves blocking the receptors or producing competitive inhibitors^6^. In this study, the protein profile of *P. mirabilis* 3059 OMPs was compared to that obtained from amikacin, phage and AMK/phage resistant variants of this strain. The bacteriophage 62A and AMK used separately did not destroy the biofilm of *P. mirabilis* 3059, while a combination of amikacin and phages was very effective in biofilm destruction (reduction even by 84 ± 3%).

The obtained results and literature data prompted us to analyse the changes in the OMPs composition due to unfavourable growth conditions (presence of amikacin and/or phages). MALDI TOF MS/MS analysis of wild type strain and all resistance variants allowed identifying the significance of proteins, which were divided into three sections: (1) occurring only in individual resistant variants and absent in the wild type strain; (2) with a significantly increased; (3) decreased intensity in resistant variants.

There were identified two interesting proteins expressed only in isolated AMKrsv: membrane fusion protein AcrA and glutamyl-tRNA synthetase. Membrane fusion protein (MFP) AcrA is a subunit of a multidrug efflux pump. In Gram-negative bacteria MFPs act as auxiliary proteins or ‘adaptors’, connecting a primary porter in the cytoplasmic membrane with an outer membrane protein, which functions as a porin or channel in the membrane. Efflux pumps are characterized by broad substrate specificity in relation to antibiotics, detergents, dyes, and organic solvents^19^. The reduction of antibiotic concentrations inside bacteria by drug efflux transporters also contributes to the development of antibiotic-specific resistance. The second protein characteristic for AMKrsv is protein glutamyl-tRNA synthetase. In *P. mirabilis* HI4320 this enzyme takes part in protein synthesis catalysing the two-step attachment of glutamate to tRNA(Glu). Germain et al.^20^ observed that phosphorylation of glutamyl-tRNA synthetase (GltX) mediated by eukaryote-like serine threonine kinase HipA caused an increase in the (p)ppGpp level leading to multidrug resistance and persistence in *E. coli* cells. On the other hand, overexpression of GltX prevented the stringent response induced by HipA and persister formation^21^.

In the group of proteins with a significantly increased expression in the AMK resistant variants, some were found to be involved in protein biosynthesis, transcription, signal transduction, which seems to correspond to well-known mechanisms of Gram-negative bacteria resistance to aminoglycosides. Some of identified proteins are usually located in cytoplasm. It was observed that, as a result of environmental stress, proteins could be exported to the cell membrane, like the elongation factor Tu (EF-Tu), which represents even 5% of the total cell proteins. It is a conserved protein, involved in translation (elongation phase) and post translation modifications, also acting as a RNA chaperone and protecting tRNA during aminoacylation^22^. Stress conditions caused by the presence of antibiotics may lead to an increased expression of EF-Tu in bacterial cells, the protein is exported to the cell membrane and plays a role of a receptor for host proteins, e.g. fibronectin, plasminogen and factor H^23^. Homologues of EF-Tu: TetO and TetM interact with the ribosome and dislodge the antibiotic from its binding site in a GTP-dependent manner^24^. Mogre et al.^25^ observed that sublethal doses of aminoglycosides cause low cost mutation in the elongation factor EF-G in *E. coli*, which confers resistance to these antibiotics. Important cytoplasmic proteins involved in antibiotic resistance are: asparaginyl-tRNA and aspartyl-tRNA synthetases and adenylosuccinate synthetase (AdSS), which are conserved enzymes performing an important function in translation and purine biosynthesis, respectively^26^. Nanduri et al.^27^ observed an increased adenylosuccinate synthetase expression in response to subinhibitional doses of antibiotics (AMX, CTC and ENR) in *Pasteurella multocida* cells. On the other hand, there are studies which suggest that AdSS could be a potential drug target in *Helicobacter pylori*^28^. Another important cytoplasmic protein—ribosomal protein L14 in *E. coli* binds directly to 23S ribosomal RNA and forms part of bridges connecting the 2 ribosomal subunits. When L14 protein interacts with RsfS, the bridge cannot be formed, and the 30S and 50S ribosomal subunits do not associate, which represses translation^29^. ATP-dependent and ATP-binding subunit HslU proteases are enzymes which operate by a variety of chemical mechanisms and are essential for bacterial cell life and pathogenicity. HslU (ClpQY) is one of ubiquitous families of intracellular proteolytic complexes in eubacteria. The others are: Lon, ClpXP and FtsH^30^. The importance of proteases in antibiotic resistance, especially to aminoglycosides has been confirmed. In *P. aeruginosa* proteases HtpX and HslVU contribute to aminoglycoside resistance. HtpX and HslVU may perform redundant back-up functions for FtsH in *P. aeruginosa* or act on disruptive polypeptides, which FtsH does not recognize. The participation of soluble as well as membrane proteases in aminoglycoside resistance indicates that some disruptive polypeptides are degraded in the cytoplasm^31^. Fernandez et al.^32^ demonstrated that mutation of protease-related proteins i.a. Lon and AsrA, proteins coded by pfpI, clpS, and clpP genes (intracellular protease mutants) affected antibiotic resistance, swarming motility and biofilm formation. The authors emphasised the importance of the regulatory function (stress response) of intracellular proteases in *P. aeruginosa*.

The overexpression of another important protease—ClpP, which could be a target for new antimicrobials has also been observed. ClpP, ATP-dependent serine peptidase, is conserved within the bacteria and plays an important role in bacterial metabolism, mainly in stress response. The inactivation of ClpP in many bacteria leads to the attenuation of important functions, for example: in *Bacillus subtilis* it causes a reduction in cell motility and sporulation, in *Staphylococcus aureus* it leads to the impairment of virulence. Low expression of that protein also results in the accumulation of stress regulators such as Spx, LexA and CtsR^33^.

During MS analysis, a number of over-produced proteins, not directly associated with antibiotic resistance, were also identified. Their overexpression could have been a result of the growth of bacteria in biofilm. The examples are protein kinases, which are responsible for the phosphorylation of proteins on serine/threonine or tyrosine residues. These enzymes are recognized as an additional signalling mechanism in prokaryotes. The virulence of some bacterial pathogens including biofilm formation also depends on the kinases^34^. Polyphosphate kinase (PPK), which shows a higher expression in AMKrsv is a highly conserved protein found in many bacteria and it is involved in the synthesis of inorganic polyphosphate (poly P) from the terminal phosphate of ATP. It has been observed in *P. aeruginosa* that mutants lacking that enzyme are deficient in motility, quorum sensing and biofilm formation^35^. Chen et al.^36^ indicated that PPK is important for the antibiotic stress response in uropathogenic *E. coli*, regulates the expression of antibiotic efflux and influx genes. PPK is also involved in *E. coli* biofilm formation. Ortiz-Severin et al.^35^ suggests that a decrease in PPK activity leads to the reduction of bacterial virulence and persistence, and increased susceptibility to antibiotics. The synthesis of PPK in bacterial pathogens could be a potential target for antimicrobial drug design. It is also very important that PPK1 homologues have not been identified in higher-order eukaryotes. Another protein which could be overexpressed in biofilm is glycerol-3-phosphate dehydrogenase (GlpD). It catalyses the conversion of glycerol 3-phosphate (G3P) to dihydroxyacetone phosphate (DHAP). In *E. coli* GlpD is localized in the cytoplasmic membrane and it has been suggested to be a component of the multidrug tolerance mechanism^37^. Overexpression of the gene for GlpD was observed in bacteria which produce increased amounts of persisters. The strain with GlpD overexpression showed a high tolerance to ampicillin and ofloxacin. The strain which did not synthesize GlpD produced fewer persisters^38^. Flagella basal body rod protein (FlgB) is a member of the flagellar motor complex. There is a hypothesis that swarming cells are more resistant to antibiotics and swarming is an effective bacterial survival strategy against antimicrobials. Butler et al.^39^ reported that swarming populations, for example *Salmonella enterica*, *P. aeruginosa*, *Serratia marcescens* and *B. subtilis*, exhibit an elevated resistance to multiple antibiotics. The survival strategy is based on living in a multi-layered colony with high bacterial density, which minimizes the exposure of bacterial cells to the antibiotic.

In our study there was only one protein—transcription termination factor NusA, which was overexpressed in Brsv. NusA is a component of the RNA polymerase elongation complex, which plays a role in transcriptional elongation, termination, anti-termination, cold shock and stress-induced mutagenesis. NusA, NusE and NusG are essential for phage lambda to alter the host RNA polymerase (RNAP) activity and to allow appreciable transcription through multiple terminators. This protein is also involved in heat shock resistance of host cells, prevents protein aggregation under heat stress conditions and has a chaperone activity (an RNA chaperone in *Mycobacterium tuberculosis* cells)^40,41^.

In the present study there were also identified proteins with a lower expression in resistant variants, which could play an important role in amikacin or phage resistance. Bacterial existence in biofilm is closely related to the resistance to aminoglycosides. Lack of sensitivity to these antibiotics is also connected with energy dependent mechanisms. The antibiotics kill metabolically active cells and the reduced metabolism of sessile bacteria has been shown to be the reason for antibiotic resistance^42^.

In AMKrsv, MoxR-like ATPase, phosphoenolopyruvate carboxykinase (PEPCK) and SucAgene product are worth attention, because a decrease in their expression could be closely related to the resistance to antibiotics. MoxR ATPases are chaperone-like proteins, widespread throughout bacteria. They facilitate the maturation of protein complexes. Wong et al.^43^ suggested that in *E. coli* MoxR ATPase from the RavA subfamily (Regulatory ATPase variant A) could play a role in aminoglycosides resistance, when it co-occurred with the protein ViaA. The authors observed that the members of MoxR ATPase interacted with highly conserved NADH: ubiquinone oxidoreductase I complex (Nuo complex), which could be identified as a mechanism of sensitisation by RavA and ViaA of *E. coli* towards aminoglycosides. Reduced expression of this protein in aminoglycosides resistant variants may confirm the role of MoxR ATPases in the resistance to these antibiotics.

Decreased intensities in resistant variants occurred also in PEPCK, an enzyme involved in glucogenesis, which catalyses the reversible conversion of oxaloacetic acid into phosphoenol pyruvic acid. It is known that the enzyme plays an important role in the pathogenesis of tuberculosis and mutations of the gene leading to reduced bacterial virulence^44^. The reduced level of the enzyme expression could be also related to the resistance to aminoglycosides. These antibiotics affect metabolically active cells, and the reduction of the metabolic rate (because of PEPCK down-regulation) may contribute to adaptive resistance against aminoglycosides. Sun et al.^42^ discovered that ten genes of *P. aeruginosa* i.a. *pckA*, coding phosphoenolpyruvate carboxykinase were downregulated in mutants exhibiting increased resistance to antibiotics e.g. norfloxacin and tobramycin. The SucA gene product is a very interesting protein which catalyses reactions from 2-oxoglutarate to succinyl-CoA in the TCA cycle. 2-oxoglutarate is an important molecule of the TCA cycle and is linked to amino acid metabolism. Shan et al.^45^ observed that the disruption of i.a. SucAB and sucCD leads to the accumulation of 2-oxoglutarate in the cells, which is related to the increased *E. coli* tolerance to gentamycin. On the other hand, it is known that sucAB genes expression decreases during biofilm formation. This enzyme is involved in cell energy metabolism. During biofilm formation and maturation the cell growth and metabolic activities decrease, so the enzyme production is reduced^46^.

In Brsv examined in our study an interesting downregulated protein—dynamin superfamily protein, was identified. The protein could play a significant role in bacterial resistance to viruses. Dynamin superfamily proteins are involved in: membrane modelling (which may contribute to masking the receptor for the virus), endocytosis and cytokinesis. It could be also probable that dynamins are synthesised to counteract osmotic stress. However, there are no significant data describing the role of dynamin proteins in phage infection^47^.

In the present study the proteins potentially participating in *P. mirabilis* antibiotic and phage resistance were identified. The lowest number of proteins with changes in their expression level were detected after the treatment of biofilm with bacteriophage. Application of phages as a second antimicrobial agent in combined therapy led to effective eradication of *P. mirabilis* 3059 biofilm and also to a reduction in the number of proteins up- and downregulated in protein profiles of AMK/Brsv compared to AMKrsv. Some of the identified proteins were cytoplasmic. Their presence among isolated OMPs could be a result of environmental stress. It was found that most of the proteins were involved in cell metabolic processes such as: proteins, purines, lipids and carbohydrates conversions. Some of them played an essential role in replication and transcription. Interestingly, chaperones and proteins involved in virulence or transport were also identified. Some of the proteins e.g. adenylosuccinate synthetase, ATP-dependent ClpP protease or polyphosphate kinase (PPK) with an increased level of expression in resistant variants are worth examining, because they may represent a target for antibacterial drugs, which could result in developing new therapies against biofilm associated *P. mirabilis* infections.

## Materials and methods

### Bacteria and bacteriophages

Uropathogenic strains of *P. mirabilis* 1281, 3059, C41 and C77 were kindly donate by the Institute “Monument—Children’s Health Centre” in Warsaw and outpatient clinic of M. Pirogow Specialist Hospital in Lodz^3^. Bacteria were stored at − 80 °C in L-Broth (BTL) supplemented with 10% DMSO (Avator). During the experiments the strains were cultivated on TSA plates for 24 h at 37 °C. Bacteriophages 39APmC32 (39A), 62Pm1984 (62), 68BPm3907 (68B), 71APm4955 (71A) were isolated from urban wastewater^48^. The phages were selected based on the results of the previous studies (data not shown). The bacterial growth inhibition after incubation with phages was studied over a period of time (0–24 h). The bacteriophages, selected for the current study, caused a significant decrease in the optical density of bacterial cultures after 6 h of incubation, while after 24 h, an increase in the bacterial population was observed, suggesting the emergence of phage-resistant variants.

### Testing the antimicrobial sensitivity of *P. mirabilis* planktonic and sessile forms

Antimicrobial sensitivity of *P. mirabilis* planktonic cells to amikacin was examined by determining the MICs (Minimum Inhibitory Concentrations) and MBCs (Minimum Bactericidal Concentrations) using the standard broth-microdilution method recommended by the Clinical and Laboratory Standards Institute^49^.

Biofilms for an antibiotic susceptibility assay were cultivated on polystyrene flat bottom microtiter plates. Bacteria were cultured in nutrient broth (NB) pH 7,1 for 24 h at 37 °C, 150 rpm, diluted (1 × 10^7^ CFU (colony forming unit)/mL) and transferred (100 µL) to each well of the plate. After 24 h incubation at 37 °C the biofilms were rinsed with 0.85% NaCl and 100 µL of a proper concentration of amikacin (AMK) was added (from 512 to 0.001 µg/mL). The plates were incubated for 24 h at 37 °C and next the biofilms were washed with 0.85% NaCl to remove unbound cells. To determine the cell viability, the biofilms were treated with MTT (Sigma). 100 µL of fresh nutrient broth and 25 µL of MTT (5 mg/mL in PBS) were added to each well of the plate. The plate was incubated for 4 h at 37 °C. Next, 150 µL of dimethyl sulfoxide and 25 µL of glycine buffer were added to dissolve formazan crystals. Absorbance was measured at λ = 550 nm. The absorbance values were proportional to the biofilm intensity. The obtained results were presented as a percentage of the reduction in absorbance in relation to the biofilm not treated with amikacin. MBIC (Minimum Biofilm Inhibitory Concentration) was determined as the antibiotic concentration causing a 50% decrease in the metabolic activity of sessile cells^15^. STATISTICA 13 (StatSoft, Poland) and nonparametric Mann–Whitney U-test comparing outcomes between two independent groups was used to determine differences between best acting agent used alone and combination of amikacin and appropriate phage (p < 0.05).

### Isolation of amikacin and/or bacteriophage resistant variants from biofilm

A *P. mirabilis* overnight culture in nutrient broth pH 7.1 diluted to a final bacterial population of 10^7^ cfu/mL was placed (100 μL) on a 96-well flat bottom plate. Biofilms were incubated for 24 h at 37 °C. Next, planktonic bacteria were removed and the wells were washed gently with 0.85% NaCl. Then, 100 μL of amikacin at a concentration of 16 μg/mL, 100 μL of bacteriophages 10^7^ PFU (plaque forming unit)/mL or 100 μL of nutrient broth containing amikacin (16 μg/mL) and phage (10^7^ PFU/mL) were added. The plate was incubated for 24 h at 37 °C. Next, the wells were washed with 0.85% NaCl, and 100 μL of nutrient broth was added to each well. Biofilms from selected wells were scraped and transferred to vials containing a medium with 16 μg/mL of amikacin or phages (1 × 10^7^ PFU/mL) or a combination of AMK and phages. After 24 h incubation at 37 °C, the cultures were placed on a solid nutrient medium containing phages, amikacin or both agents. Growth intensity on liquid selective medium—LSM (turbidity) and solid selective medium—SSM (number of colonies) was marked as: 0—no growth, 1—weak growth, 2—medium growth, 3—intensive growth. The amikacin (AMKrsv) or bacteriophage (Brsv) or AMK and phage (AMK/Brsv) resistant variants were isolated. For each randomly isolated resistant variant, the values of MIC, MBC and/or RTD (routine test dilution, defined as the highest phage dilution producing a reaction of confluent or semi-confluent lysis on an appropriate indicator strain) were determined. The amikacin MICs and MBCs were evaluated as described above. The RTD value was determined by spotting ten-fold dilutions of the phage in broth onto an agar surface inoculated with a broth culture of a wild type strain (positive control) and resistant variants of *P. mirabilis*.

The effect of AMK, phages and the agents used in a combination on biofilms was also evaluated using the MTT test described above.

### Outer membrane proteins extraction

The OMP extraction of the wild type (WT) *P. mirabilis* strain 3059 and the obtained resistant variants was performed using the method described by Piccini et al*.*^50^. Bacteria were centrifugated (4000 rpm for 20 min., 22 °C) and the pellets were washed twice with 10 mM Tris–HCl buffer (pH 8). Next, bacterial cells were disrupted by sonication, and cell debris was removed by centrifugation (4000 rpm for 30 min., 4 °C). The obtained supernatants were recentrifuged at 14,500 rpm for 45 min. at 4 °C, next the pellets were suspended in 10 mM Tris-HCI buffer pH 8.0, containing MgCl_2_ (10 mM) and Triton X-100 (2% v/v). After incubation (45 min, 37 °C) the samples were centrifuged at 14,500 rpm for 45 min. at 4 °C and the pellets were suspended in ultra-pure water. The proteins concentrations were analysed using the Bradford method^51^.

### Proteomic analysis

Outer membrane proteins analysis was performed as previously described^52^. First, the OMPs were separated in 2D gel electrophoresis in triplicates. Gels stained with Coomassie Brilliant Blue G-250 were scanned using a Power-Look 1000 (UMAX Technologies Inc., Dallas, TX), and were analysed by PDQuest version 7.3.0 (Bio-Rad Laboratories).

Protein spots were excised from gels, successively destained with aqueous 50% acetonitrile (pH 8.0) containing 25 mM ammonium bicarbonate for 20 min. at 50 °C and washed with distilled water. The destained gel discs were dehydrated twice using 85 μL 100% acetonitrile for 3 min. at room temperature. Next, the gel fragments were dried at room temperature, swollen with 10 μL 25 mM ammonium bicarbonate containing 50 ng trypsin (Promega, sequencing grade) and incubated overnight at 37 °C. The 0.3 μL peptides were mixed with an equal volume of a saturated solution of α-cyano-4-hydroxycinnamic acid in 50% acetonitrile containing 0.1% trifluoroacetic acid, applied to a sample plate and dried at room temperature. Next, proteins were analysed by MALDI-TOF MS using a 4700 Proteomics Analyzer (Applied Biosystems). Proteins were identified by automated peptide mass fingerprinting using the Global Proteome Server Explorer software 3.0 (Applied Biosystems). The identified proteins had MASCOT report protein scores for MS or total ion scores for MS/MS with confidence intervals (C.I.) greater than 95%. Searches for sequence similarity were performed by BLAST against the NCBI.

### Ethics statement

The bacterial strains used in the study are part of the collection of Department of Biology of Bacteria, University of Lodz and were collected and kindly donated by the Institute “Monument—Children’s Health Centre” in Warsaw, and outpatient clinic of M. Pirogow Specialist Hospital in Lodz. The strains were collected before 2002 in accordance with methods and procedures laid down in the Polish provision in force at the time. All methods were carried out in accordance with relevant guidelines and regulations.

## Supplementary Information

Supplementary Figure 1.
