# Supplementary material for: Amikacin and bacteriophage treatment modulates outer membrane proteins composition in Proteus mirabilis biofilm
Source: Sci Rep. 2021 Jan 15;11:1522. doi: 10.1038/s41598-020-80907-9 (PMC7810710; doi:10.1038/s41598-020-80907-9)
Supplement: Supplementary file 1 — Supplementary Figure 1. [file 41598_2020_80907_MOESM1_ESM.docx]

Amikacin and bacteriophage treatment modulates outer membrane proteins composition in *Proteus mirabilis* biofilm

Authors: Agnieszka Maszewska^1^, Magdalena Moryl^1^, Junli Wu^2^, Bin Liu^2^, Lu Feng^2^, Antoni Rozalski^1^

^1^Department of Biology of Bacteria, Institute of Microbiology, Biotechnology and Immunology, Faculty of Biology and Environmental Protection, University of Lodz, Banacha 12/16, 90-237 Lodz, Poland

^2^TEDA Institute of Biological Sciences and Biotechnology, Nankai University, Tjanjin, P.R.China

Corresponding author’s e-mail: magdalena.moryl@biol.uni.lodz.pl


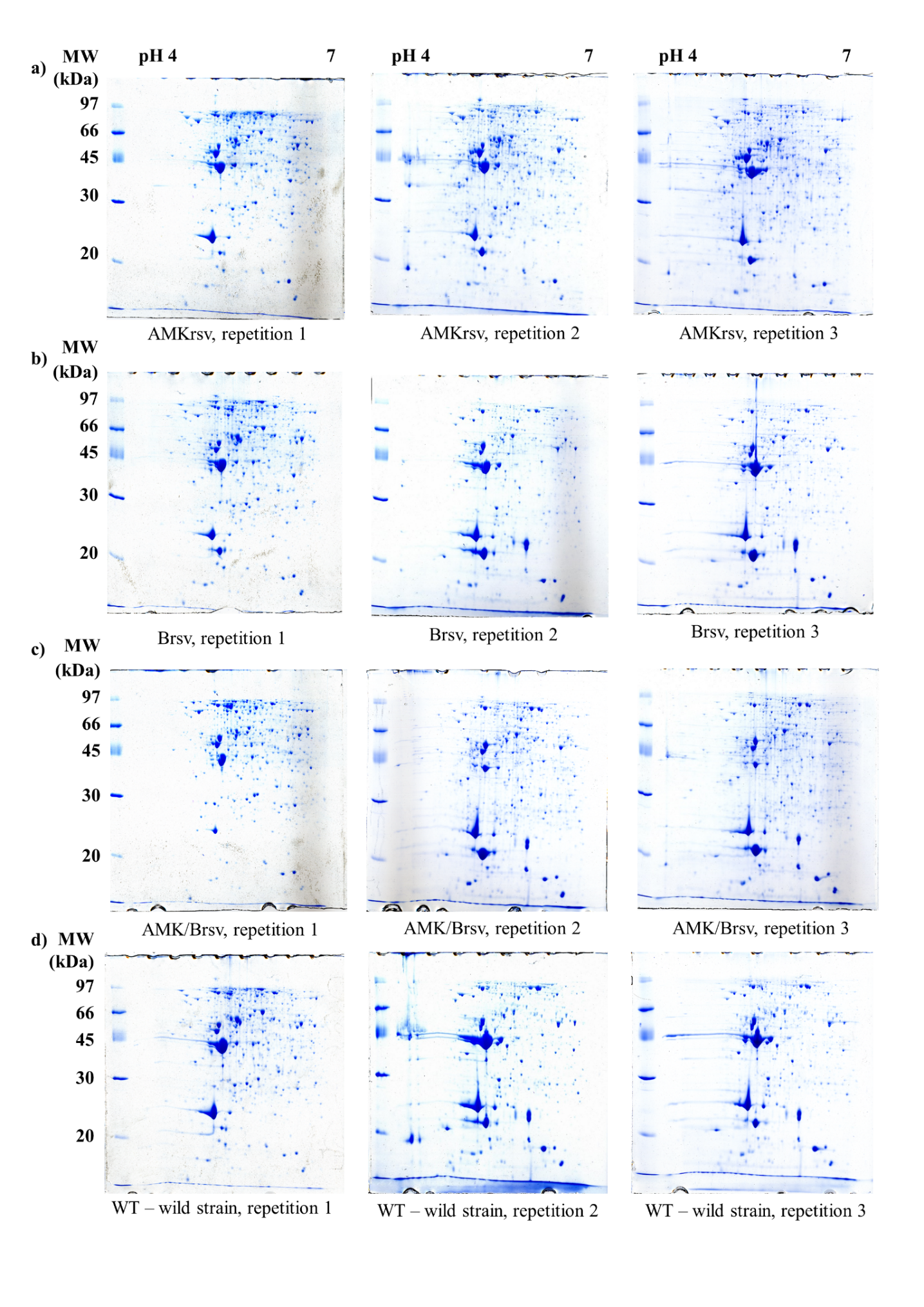


**Supplementary Figure 1.** 2D gel electrophoresis images of *Proteus mirabilis* 3059 OMPs: a) amikacin resistant variant (AMKrsv), b) bacteriophage resistant variant (Brsv), c) amikacin and bacteriophage resistant variant (AMK/Brsv) and d) wild type strain.

Proteins were stained with Coomassie Brilliant Blue G-250, gels were scanned using a Power-Look 1000 (UMAX Technologies Inc., Dallas, TX).
